# Removal of an infant's gastric duplication cyst through endoscopic submucosal dissection: A case report

**DOI:** 10.1097/MD.0000000000014820

**Published:** 2019-03-22

**Authors:** Ying Fang, Tianjiao Gao, Hongbin Yang, Shiyang Ma, Quanlin Li, Ping-Hong Zhou

**Affiliations:** a Department of Gastroenterology, The Affiliated Children Hospital of Xi’an Jiaotong University; b Department of Gastroenterology, the Second Affiliated Hospital of Xi’an Jiaotong University, Xi’an, Shaanxi; c Zhongshan Hospital, Fudan University, Shanghai, China.

**Keywords:** endoscopic submucosal dissection, gastric duplication cyst, pediatric

## Abstract

**Rationale:**

Gastric duplication cyst is an anomaly that primarily occurs to children. Apart from the conventional use of surgical resection, few cases using endoscopic treatment have been reported.

**Patient concerns:**

A 5-month-old female infant was hospitalized with the chief complaint of gastric cyst. No significant abnormalities were identified by physical examination.

**Interventions:**

Endoscopic submucosal dissection (ESD) was performed successfully for the infant and the duration was less than 20 minutes. The patient showed no postoperative complications.

**Outcomes:**

At 4 months during the follow-up, upper endoscopy revealed a small scar at the previous site of the lesion and no recurrence.

**Lessons:**

According to the results of PUBMED review, she was the youngest with gastric duplication cyst removed with ESD. The less invasive ESD should be considered an effective therapeutic option to remove gastric duplication cyst in children.

## 1 Introduction

Accounting for 2% to 7% of all gastrointestinal duplications, gastric duplication cyst is an anomaly that primarily occurs to children and rarely to adults.^[1]^ Currently, the treatments reported in the literature include surgical resection and laparoscopic surgery in newborns.^[2,3]^ Few cases on endoscopic treatment have been reported.^[4]^ Endoscopic submucosal dissection (ESD), an minimal invasive technique, can achieve a high rate of en bloc resection and reduce the pain after surgery for patients with early upper and lower gastrointerology trace neoplasia.^[4]^ Eom et al^[5]^first reported a gastric duplication cyst that had been resected completely by ESD in a 28-year-old man in 2011. However, ESD resection of gastric duplication cysts in newborns is rarely reported. This case report was about a 5-month-old female infant whose gastric duplication cyst was removed with ESD without any complication. According to the results of PUBMED review, she was the youngest with gastric duplication cyst removed with ESD.

## 2 Case report

**Figure 1:**
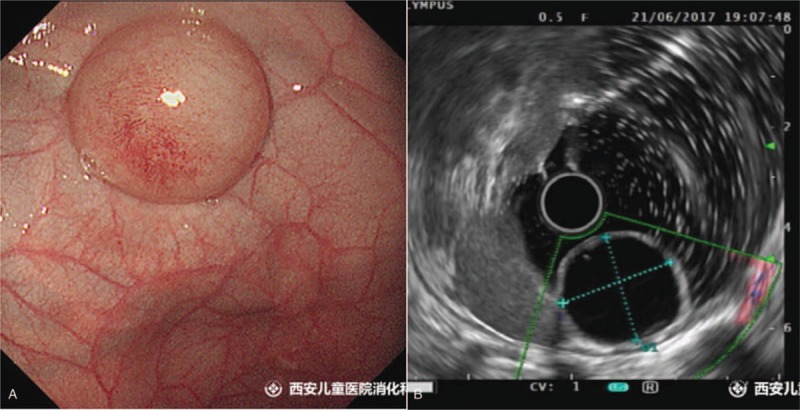
A, Upper endoscopy showed bulging lesions. B, Endoscopic ultrasonography showed that the lesion originated from the submucosal layer.

**Figure 2:**
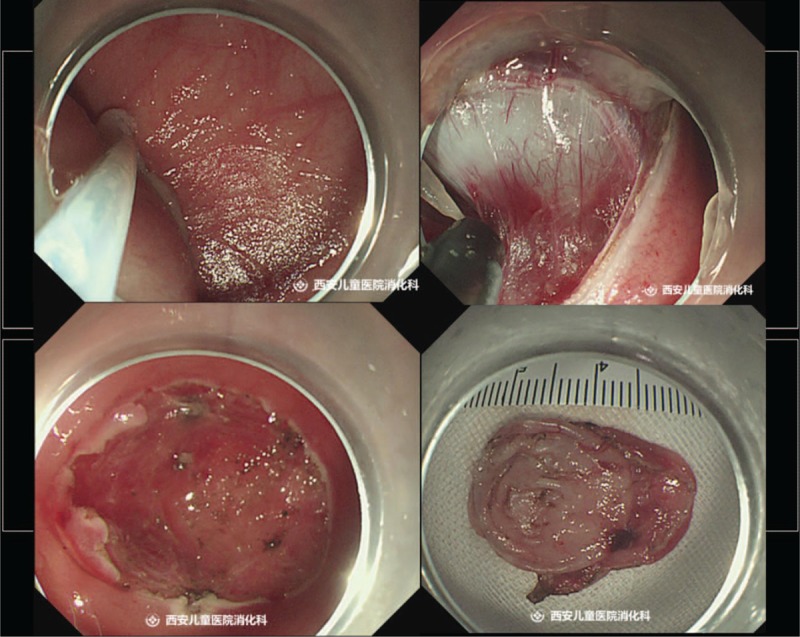
Endoscopic submucosal dissection of gastric duplication cyst.

A 5-month-old female infant was hospitalized with the chief complaint of gastric cyst in the fetal period. Six months before, the patient was diagnosed of intragastric cysts as her mother did prenatal examination in the local hospital. The child at birth was asymptomatic, without any vomiting, abdominal pain, abdominal distension, and other discomfort. Physical and laboratory examination showed she had no obvious abnormalities after being referred to our hospital. Upper endoscopy revealed an ellipsoid submucosal polyp at gastric fundus (Fig. 1A). Endoscopic ultrasonography showed an anechoic homogenous, oval lesion (22.2 mm × 21.2 mm) originating from the submucosal layer (Fig. 1B). There was no blood flow in the lumen. Vascular echo was detected in the proximal base of the wall. Blood flow was not found in the apical. Follow-up was recommended, but the infant's parents insisted on the immediate surgical removal and ESD was performed (Fig. 2).

**Figure 3:**
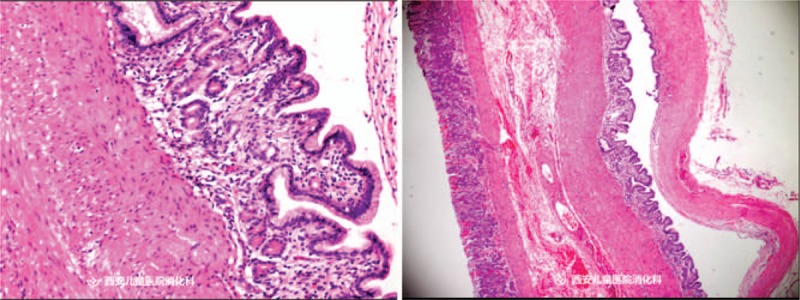
Histopathologic features of the resected specimen (left: original magnification ×10; right: original magnification ×4). Pathologic examination revealed that the submucosal mass was a cystic lesion lined with a mucosa of columnar epithelium.

**Figure 4:**
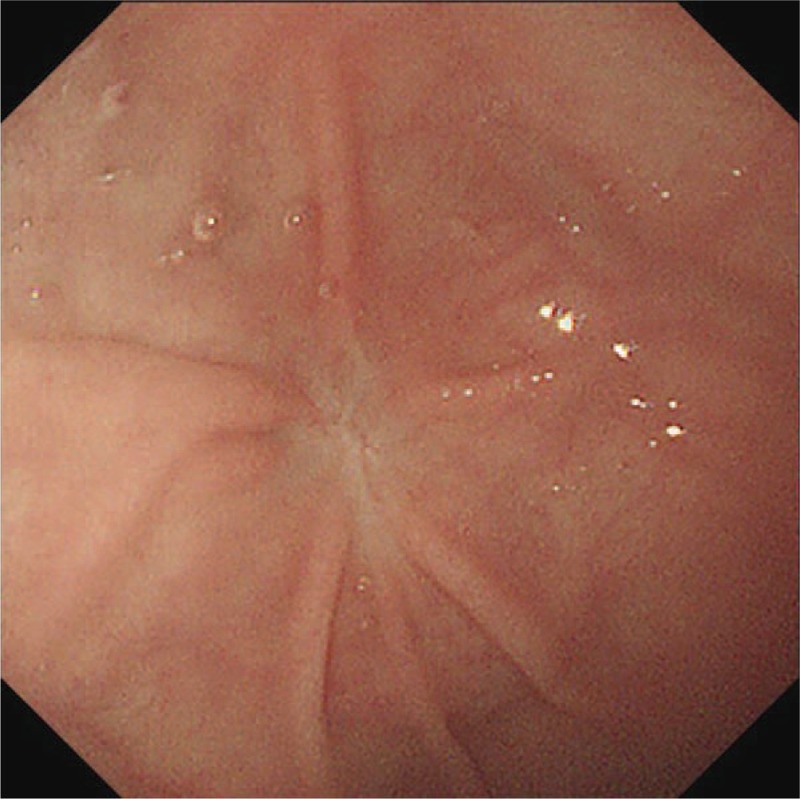
Upper endoscopy 4 months later revealed a small scar at the previous site of the lesion without recurrence.

When the child was in general anesthesia, upper endoscopy revealed a polyp at gastric fundus. The disposable OLYMPUS snared over the base. It was impossible to remove the lesion via electrocoagulation due to the wide base. OLYMPUS IT knife was used to peel off the lesion base (6 times of cutting). Biopsy forceps were used to stop the bleeding in the wound at the lesion base (10 times of clamping). Histopathologic examinations revealed a cystic lesion (2.0 cm × 2.0 cm) lined with a mucosa of columnar epithelium. Gastric duplication cyst was confirmed (Fig. 3). A 10 # gastric tube was placed and externally fixated by 30 cm. Upper endoscopy was withdrawn after the gas was inhaled into the stomach. The operation was successful and the duration was less than 20 minutes. At 4 months after the surgery, upper endoscopy revealed a small scar at the previous site of the lesion and no recurrence (Fig. 4).

## 3 Discussion

The first case report of gastric duplication cyst was published by Wendel in 1911. Duplication cysts are described as spherical or tubular. The spherical cyst is more common (accounting for 82% of all duplication cysts) and does not communicate with the bowel lumen. The tubular cyst is commonly seen in the large and small bowels and communicates with the bowel lumen.^[6]^ Gastric duplication cyst can be found anywhere in the stomach, but the distal greater curvature is most vulnerable. In the gastrulation phase, duplication cyst develops from an endomesenchymal tract between the yolk sac and the amnion. Several theories have attempted, but all failed to explain the embryological development of gastric duplication cyst.^[7]^

The essential criteria for diagnosing a gastric duplication cyst are: the wall of the cyst is contiguous with the stomach wall; the smooth muscle in the cyst is continuous with that of the stomach; the cyst wall is lined with gastric epithelium or other types of gut mucosa.^[8]^

The clinical presentation of gastric duplication cyst is examined based on its size and position. Most patients are asymptomatic and the cyst is discovered incidentally by radiological examination or upper endoscopy. However, the patients may later show symptoms like epigastric pain, epigastric fullness, vomiting, weight loss, dysphagia, dyspepsia, and anemia. In the physical examination, a palpable abdominal mass is most indicative. Occasionally, complications arise, such as gastric outlet obstruction, gastric perforation.^[9]^

Close observation and follow-up are usually recommended for small and asymptomatic duplication cyst. To remove a gastric duplication cyst, conventional treatments include surgical resection, minimal invasive surgery, and laparoscopic treatment.^[6]^ ESD is an emerging technique characterized with less wound pain, lower cost, decreased hospital stay, quicker postoperative recovery, and less wound-related complications. Eom et al^[5]^ first reported the case of a complete resection of gastric duplication cyst by ESD in 2011. However, ESD is still little used to remove gastric duplication cyst, especially for infants.

The patient in this case was only 5 months. Gastric cyst was found at week 40 in the gestational period. The patient's parents insisted on the removal and ESD was performed. The operation was successful without any complication. The operating time was less than 20 minutes. Histopathologic examinations confirmed the diagnosis of gastric duplication cyst.

We believe that ESD is a minimal invasive and safe treatment that can completely remove children's gastric duplication cyst. ESD can be considered an effective therapeutic option to remove gastric duplication cyst.

## Author contributions

**Conceptualization:** Ping-Hong Zhou.

**Resources:** Tianjiao Gao, Hongbin Yang.

**Supervision:** Shiyang Ma, Quanlin Li.

**Validation:** Ying Fang, Ping-Hong Zhou.

**Writing – original draft:** Ying Fang.

**Writing – review & editing:** Ping-Hong Zhou.
